# Design, Synthesis and Biophysical Characterisation of CPP‐PNA Conjugates for Targeting Oncogenic microRNA‐221

**DOI:** 10.1002/psc.70129

**Published:** 2026-10-02

**Authors:** Martina Rotondo, Françoise Illien, Fabienne Burlina, Barbara Biondi

**Affiliations:** ^1^ University of Naples Federico II Naples Italy; ^2^ Padova Unit Institute of Biomolecular Chemistry of CNR Padova Italy; ^3^ Sorbonne Université, École Normale Supérieure, Université PSL, CNRS, Chimie Physique et Chimie du Vivant, CPCV Paris France

**Keywords:** antisense therapy, cell‐penetrating peptides (CPPs), click chemistry, disulfide bridge, miRNA‐221, peptide nucleic acids (PNAs), SPPS

## Abstract

Peptide nucleic acids (PNAs) are a promising platform targeting disease‐associated microRNAs due to their high binding affinity, excellent sequence specificity and resistance to enzymatic degradation. However, their therapeutic application is often limited by inefficient cellular uptake. In this study, we present the design, synthesis and physicochemical characterisation of cell‐penetrating peptide (CPP)‐PNA conjugates that target the oncogenic microRNA miRNA‐221‐5p, as well as their preliminary biological evaluation. Anti‐miRNA‐221‐5p PNAs functionalised for conjugation with CPPs via Cu(I)‐catalysed azide‐alkyne cycloaddition (CuAAC) or disulfide‐mediated coupling were synthesised using solid‐phase peptide synthesis. Circular dichroism and UV‐melting analyses confirmed the formation of a stable, sequence‐specific PNA‐RNA heteroduplex with miRNA‐221‐5p. This demonstrated strong hybridisation and high thermodynamic stability compared to scrambled control sequences. Cell proliferation assays indicated modest but reproducible antiproliferative effects for the CPP‐PNA conjugates co‐incubated with chloroquine, with no evidence of acute cytotoxicity. These findings emphasise the potential of CPP‐PNA systems for the intracellular delivery of antisense agents and highlight the importance of the linker nature in determining their biological performance.

## Graphical abstract

Cell‐penetrating peptide‐peptide nucleic acid (CPP‐PNA) conjugates that target the oncogenic microRNA‐221‐5p were synthesised using Cu(I)‐catalysed azide‐alkyne cycloaddition (CuAAC) and disulfide‐mediated conjugation strategies. These constructs form highly stable, sequence‐selective PNA/RNA heteroduplexes and demonstrate preliminary antiproliferative activity in A549 lung cancer cells when endosomal escape is enhanced.

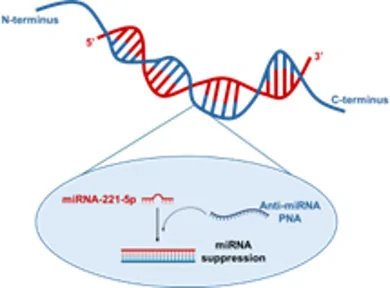

## 1 Introduction

MicroRNAs (miRNA) are small, noncoding RNAs comprising approximately 17–22 nucleotides [1]. They regulate gene expression at the post‐transcriptional level by guiding the RNA‐induced silencing complex (RISC) to complementary sequences within target messenger RNAs (mRNAs) [2, 3]. This mechanism enables miRNAs to modulate a wide range of biological processes, including cell proliferation, differentiation, apoptosis and development [4, 5]. Dysregulation of miRNA expression is associated with the onset and progression of many human diseases, particularly cancer, where miRNAs can act as tumour suppressor or oncogenic regulators (oncomiRNAs) [6, 7].

One such oncogenic microRNA is miRNA‐221, which has emerged as a key regulator of tumour progression in several epithelial malignancies [8]. It belonged to the miRNA‐221/222 cluster and is frequently overexpressed in solid tumours, including non–small‐cell lung cancer (NSCLC) [9]. Aberrant expression of miRNA‐221 contributes to malignant transformation by repressing multiple tumour suppressor genes, such as PTEN, CDKN1B (p^27[Kip1]), CDKN1C (p57^[Kip2]) and SOCS family proteins. This activates prosurvival signalling pathways, including PI3K/Akt and JAK/STAT [9, 10, 11]. Furthermore, miRNA‐221 promotes angiogenesis, epithelial–mesenchymal transition (EMT), metastatic dissemination and resistance to chemotherapy. These properties identify miRNA‐221 as a promising therapeutic target for nucleic acid‐based anticancer strategies [9].

Several antisense technologies have been explored for the inhibition of oncogenic miRNAs, including modified oligonucleotides, locked nucleic acids (LNAs) and small interfering RNAs (siRNAs) [12, 13]. However, these approaches are often limited by issues relating to stability, specificity and intracellular delivery. Peptide nucleic acids (PNAs) represent an attractive alternative antisense platform [14]. PNAs are synthetic nucleic acid analogues in which the natural sugar‐phosphate backbone is replaced by a neutral polyamide scaffold composed of N‐(2‐aminoethyl)glycine units (Figure 1) [15]. This unique structure gives them a high binding affinity for complementary DNA or RNA sequences, exceptional resistance to nucleases and proteases and improved chemical stability [16, 17, 18]. Consequently, PNAs are being investigated increasingly as potent inhibitors of gene expression and miRNAs activity.

**FIGURE 1:**
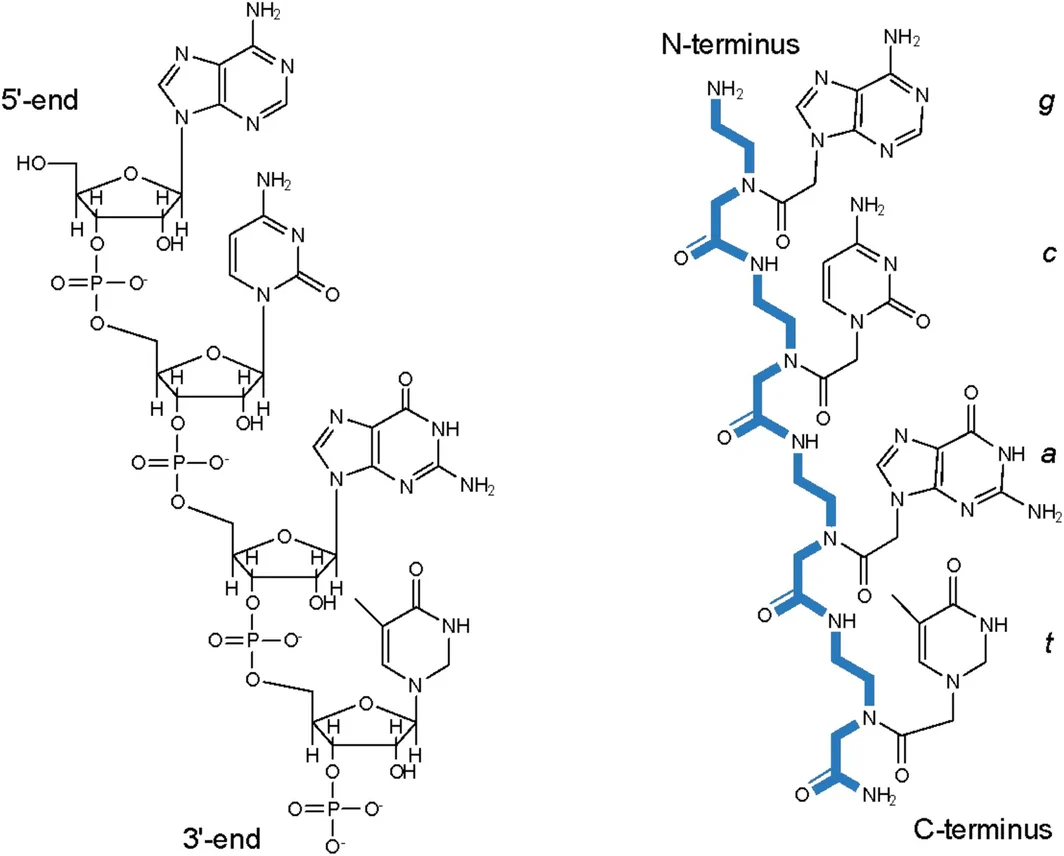
A comparison of the structure of conventional nucleic acids and PNAs.

The use of PNAs as anti‐miRNA agents has evolved considerably over the past two decades. Early studies demonstrated that antisense PNAs could effectively inhibit oncogenic miRNAs and modulate downstream gene expression, thus providing proof for PNA‐mediated miRNA targeting [19, 20]. Subsequent investigations have expanded these applications to different disease models and delivery strategies, demonstrating the versatility of PNAs as highly stable antisense molecules [21, 22]. More recently, advances in PNA chemistry and conjugation methodologies have enabled the increasingly efficient modulation of disease‐associated miRNAs, further reinforcing the potential of PNA‐based therapeutics for precision medicine [23].

However, their therapeutic application is currently limited by poor cellular uptake, primarily due to the absence of charged groups in the backbone and low intrinsic membrane permeability [24, 25]. Efficient intracellular delivery remains one of the major barriers to therapeutic application of PNAs. Several delivery platforms have therefore been investigated, including cationic lipids [26], lipid nanoparticles (LNPs) [26], cationic polymers [27] and ligand‐mediated approaches based on antibodies [28] or aptamers [28, 29]. Although lipid‐based systems generally provide high transfection efficiency, they may suffer from formulation complexity, endosomal trapping and dose‐dependent toxicity [30]. In contrast, cationic polymers are frequently associated with cytotoxicity and limited biocompatibility [31]. Antibody‐ and aptamer‐mediated strategies offer high target specificity, but they require more complex chemical conjugation and are often restricted to cells that express the corresponding receptor [32]. By contrast, cell‐penetrating peptides (CPPs) are a versatile delivery platform as they can be directly conjugated to antisense cargoes and are compatible with different chemical architectures [33, 34, 35]. CPPs are short peptides, typically cationic, that can cross biological membranes and facilitate the intracellular transport of various cargo molecules, including nucleic acids, proteins and nanoparticles [36, 37]. Through mechanisms involving direct membrane translocation and/or endocytosis, CPPs have demonstrated considerable potential as delivery vectors for antisense agents and other therapeutic macromolecules [38, 39]. The modular nature of CPPs also enables the optimisation of the peptide sequence, linker chemistry and cargo release, making them particularly attractive for the intracellular delivery of PNA therapeutics.

Several studies have demonstrated the effectiveness of CPP‐mediated delivery of antisense PNAs for gene regulation and miRNA targeting. CPP‐PNA conjugates have successfully enhanced intracellular delivery and biological activity in various experimental models, emphasising the importance of selecting appropriate peptides and employing effective conjugation strategies to achieve efficient cytosolic delivery [35]. Recent studies have also demonstrated the applicability of CPP‐mediated delivery to anti‐miRNA PNAs and other therapeutic nucleic acid cargoes, supporting their use as a promising alternative to conventional transfection reagents [34].

Building on previous studies, this work focuses on developing CPP‐PNA conjugates that target the oncogenic miRNA‐221‐5p. While previous reports mainly demonstrated proof‐of‐concept delivery, our study combines two complementary conjugation chemistries (CuACC click chemistry and disulfide‐mediated coupling) with detailed physicochemical characterisation. This includes circular dichroism and thermal denaturation studies, as well as preliminary biological evaluation. This integrated approach enables us to investigate both intracellular delivery and how linker chemistry influences the stability and biological performance of CPP‐PNA conjugates. This provides additional insights for the rational design of PNA‐based therapeutics.

## 2 Materials and Methods

### 2.1 Materials

All Fmoc‐protected amino acids, Rink amide resin, 2‐chlorotrityl chloride resin, coupling reagents (HBTU, HOBt, DIC, Oxyma Pure), piperidine, DBU and analytical‐grade solvents were purchased from commercial suppliers and used without further purification.

The synthetic miRNA‐221‐5p RNA oligonucleotide used for biophysical studies was purchased from Eurogentec (Belgium).

Cell culture reagents were obtained from Thermo Fisher Scientific unless otherwise specified.

### 2.2 PNA Synthesis

PNA oligomers were synthesised on a 0.075 mmol scale using the standard PNA protocol. The synthesis was performed on a Fmoc‐Rink‐Amide resin (0.5 mmol/g). Fmoc deprotection was initially performed using 20% piperidine in DMF. To minimise aggregation during the synthesis, the deprotection protocol was modified from the 14th monomer onward by using a solution of 5% (w/v) piperazine in DMF containing 2% DBU. For the coupling step, two equivalents of PNA monomers were dissolved in DMF; a solution of Oxyma Pure and DIC in DMF (2 eq) was added. Each coupling reaction was allowed to proceed for 75 or 90 min in the case of guanine monomer. The PNA oligomer was cleaved from the resin and deprotected by a treatment with a solution of TFA/H_2_O/TIS (95/2.5/2.5 v/v/v) or TFA/H_2_O/DODT/TIS (94/1/2.5/2.5 v/v/v/v), if a cysteine was present, for 180 min at room temperature. The filtrate was flushed by a stream of nitrogen to remove most of TFA, and the PNA was precipitated by addition of cold diethyl ether, washed three times with diethyl ether and dried in vacuo. Purification was carried out by semipreparative RP‐HPLC applying a linear gradient of acetonitrile (0.05% TFA) from 10% to 40% in 30 min on a Luna C18, 300Ȧ, 10‐μm column. PNA purity and identity were confirmed by LC‐ESI‐MS system applying a gradient 5%–95% of acetonitrile in 30 min on a Phenomenex Kinetex C18, 3.5 μm, 100Ȧ, 4.6 × 100 mm column. PNA was identified by electrospray mass analysis.

### 2.3 Peptide Synthesis

CPPs were synthesised using Syro Wave (Biotage) peptide synthesiser on a 0.1625 mmol scale using the standard protocol. The synthesis of the azide‐bearing CPPs was performed on a Fmoc‐Rink‐Amide resin (0.65 mmol/g). Cys‐containing CPPs were elongated on 2‐chlorotrityl chloride (2‐CTC) resin (1.6 mmol/g). Fmoc deprotection was performed using 20% piperidine in DMF. For the coupling step, three equivalents of amino acid were dissolved in DMF; a solution of HBTU/HOBt in DMF (3 eq) in presence of 6 eq of DIPEA was added. Double coupling steps were applied to each amino acid residue to ensure efficient chain elongation. The peptide was cleaved from the resin and deprotected by a treatment with a solution of TFA/H_2_O/TIS (95/2.5/2.5 v/v/v) and the activator 2,2‐dithiopyridine, if a cysteine was present, for 120 min at room temperature. The filtrate was flushed by a stream of nitrogen to remove the most of TFA, and the peptide was precipitated by addition of cold diethyl ether, washed three times with diethyl ether and dried in vacuo. Purification was carried out by semipreparative RP‐HPLC applying a linear gradient of acetonitrile (0.05% TFA) on a Luna C18, 300Ȧ, 10‐μm column. Peptides purity and identity were confirmed by LC‐ESI‐MS system applying a gradient 5%–95% of acetonitrile in 30 min on a Phenomenex Kinetex C18, 3.5 μm, 100Ȧ, 4.6 × 100 mm column. Peptides were identified by electrospray mass analysis.

### 2.4 CuACC Conjugation

Alkyne‐functionalised PNAs (1 eq) and azide‐functionalised CPPs (1.3 eq) were dissolved in a mixture of water and *tert*‐butanol (1:3, v/v). CuSO_4_·5H_2_O (4 eq) and sodium ascorbate (16 eq) were added to generate Cu(I) in situ. The reaction mixture was stirred at room temperature for 24 h. The resulting conjugates were purified by RP‐HPLC and characterised by analytical HPLC and ESI‐MS.

### 2.5 Disulfide Coupling

Disulfide‐linked conjugates were prepared by thiol‐disulfide exchange between cysteine‐functionalised PNAs (1 eq) and CPPs containing an activated cysteine residue (1 eq). Reactions were performed in ammonium acetate buffer (pH 4.5) at room temperature. The resulting conjugates were purified by RP‐HPLC and characterised by analytical HPLC and ESI‐MS.

### 2.6 Conformational Studies by CD Spectroscopy

CD spectra were recorded on a Jasco‐1500 spectrophotometer using Spectra Manager (Jasco) software. CD spectra were recorded at 25°C using a 1‐cm quartz cell, a 320‐ to 200‐nm measurement range, 50 nm/min of scanning speed, 1‐nm bandwidth, 4‐s response time, 0.5‐nm data pitch and are the results of four scans. The measurements are taken in duplicate.

### 2.7 UV‐Melting Analysis

UV measurements were carried out on an IMPLEN NP80 UV/Vis spectrophotometer by using 1‐cm quartz cells of 1 mL of internal volume. Single‐strand PNA and complementary RNA were dissolved in ultrapure water and were annealed by warming up at 95°C and slowly cooling down to 15°C in phosphate buffer 10 mM, 10 mM of NaCl, pH = 7.4. The thermal denaturation experiment was carried out using 2.5‐μM duplex in phosphate buffer 10 mM, 10 mM of NaCl, pH = 7.4 at a 0.5°C/min scan speed, recording at 260 nm every 5°C. The measurements are taken triplicate.

### 2.8 Cell Culture

A549 cells were cultured in DMEM medium supplemented with 10% fetal bovine serum (FBS), penicillin (100,000 IU/L), streptomycin (100,000 IU/L) and amphotericin B (1 mg/L) in a humidified atmosphere containing 5% CO_2_ at 37°C.

### 2.9 Cell Proliferation Assay

Cell proliferation was determined with the Cell Counting Kit 8 (CCK‐8) from Dojindo Molecular Technologies. 96‐well plates were inoculated with 100 μL/well of a suspension of A549 cells (2000 cells/well). After 24 h of incubation (37°C, 5% CO_2_), 10 μL of the peptide solution (0; 2.5; 5; μM final) was added, either alone or in the presence of chloroquine (150 μM) or calcium ions (5 mM), and the plate was further incubated for 1, 24 and 48 h. During the incubation period, the cells were supplemented daily with FBS. After washing, the cells were incubated with 100 μL of 10% CCK‐8 in DMEM for 3 h, and the absorbance was measured at 450 nm with a microplate reader (Polarstar Optima). Controls corresponded to untreated cells (negative control, 100% viability) and cells treated with 0.2% of Triton X‐100 (positive control, 0% viability).

### 2.10 Quantification by Fluorometry of the Efficiency of Cellular Uptake of the CPP‐PNA Conjugates

For internalization quantification assay, 500,000 A549 cells/well are seeded in a 12‐well plate the day prior to the experiment. After 24 h of incubation (37°C, 5% CO2) one million adherent A549 cells were incubated 1 h at 37°C with CF‐CPP‐PNA conjugates (5 μM) in 1 mL of DMEM glutamax. At the end of the incubation time, the supernatant was removed and cells were washed with HBSS at room temperature. A reduction step with TCEP was then performed for conjugates linked via a disulfide bridge: 200 μL/well of TCEP solution (2 mM in 50 mM TRIS buffer, pH 7.5) was added and incubated for 1.5 min at room temperature (this TCEP treatment was omitted for the triazole‐linked conjugates). After washing cells with HBSS, (0.05%) 500 μL of trypsin/EDTA (37°C, 5 min) was added for 5 min to digest the remaining extracellular and membrane‐bound species and to detach cells. After addition of enzyme inhibitors (100 μL of one) mixed with 100 μL of bovine serum albumin (1 mg/mL), cells were transferred into a microtube, centrifuged, washed with 1 mL of 50 mM of Tris buffer pH 7.4, 0.1% BSA and lysed in 200 μL 50 mM of Tris pH 7.4, 1 M of NaCl, 1% NP40 (for the disulfide conjugates, 2 μL of TCEP solution [0.5 M, pH 7] was added to the lysis buffer). The samples were then sonicated for 30 min and centrifuged 10 min at 16,000 g. About 100 μL of the supernatant from each sample was dispensed into a black 96‐well plate. The fluorescence intensity (excited at 488 nm) of the samples was measured using a microplate reader (Polarstar Optima). To obtain the amount of the internalised species, samples for calibration curves were prepared in parallel. For this, 10 different amounts (from 2 to 500 pmoles) of the appropriate CF‐CPP conjugate were added in the lysis buffer; 200 μL was added to lysates cells containing one million A549 cells. Cells were lysed, and the fluorescence intensity was measured as described before. The amounts of internalised species were calculated by comparing the fluorescence intensity of the sample with the calibration curve. Noteworthy, this protocol allows the absolute quantification of the internalised fluorescent species (all compartments included).

### 2.11 Statistical Analysis

Data are presented as mean ± standard deviation (SD). Biological assays were performed in two independent biological experiments, each carried out with three replicates for each experimental condition. Statistical analyses were performed using one‐way ANOVA followed by Turkey multiple comparison test. Statistical significance was defined as *p* < 0.05.

## 3 Results and Discussion

### 3.1 Design and Synthesis of Anti‐miRNA‐221‐5p PNA Constructs

To develop peptide‐PNA conjugates that can modulate the oncogenic miRNA‐221‐5p, an antisense PNA sequence that is complementary to the miRNA was designed and synthesised using standard Fmoc‐based solid‐phase peptide synthesis (SPPS) (Table 1, Entry 2). The PNA sequence was assembled manually using Oxyma Pure/DIC (N,N′‐diisopropylcarbodiimide) [40, 41, 42] as the coupling reagents to ensure efficient activation. Two lysine residues were incorporated at the C‐terminus to improve aqueous solubility and reduce the aggregation behaviour commonly associated with PNA oligomers. Additionally, a 5‐hexynoic acid moiety was introduced at the N‐terminus to provide terminal alkyne functionality, which is suitable for subsequent Cu(I)‐catalysed azide‐alkyne cycloaddition (CuCAAC) reactions involving azide‐functionalised CPPs.

**TABLE 1:** miRNA‐221‐5p and PNAs sequences considered in this study.

Compound	Sequence
miRNA‐221‐5p	5′‐ACCUGGCAUACAAUGUAGAUUU‐3′
Anti‐miRNA‐221‐5p PNA	H‐aaatctacattgtatgcc‐KK‐NH_2_
Scrambled PNA	H‐gtctttacagccatataa‐KK‐NH_2_

During initial synthesis attempts, LC‐ESI‐MS monitoring revealed increased formation of deletion sequences and side products as the oligomer length increased. This behaviour is consistent with previously reported aggregation phenomena in PNA synthesis, which are caused by strong nucleobase stacking interactions between growing chains on the resin. To address these limitations, the synthetic protocol was optimised by introducing a modified deprotection strategy based on piperazine/DBU [43] and by performing reactions under high dilution conditions in DMF. Coupling times were also extended, particularly for sterically demanding nucleobases such as guanine. These modifications significantly improved the overall yield and purity of the crude PNA, as confirmed by analytical HPLC profiles.

Following cleavage of the oligomers from the solid support and purification by preparative RP‐HPLC, the identity of the alkyne‐functionalised anti‐miRNA‐221‐5p PNA was confirmed by ESI‐MS analysis (Figure S1), which showed multiply charged ions corresponding to the expected molecular mass. The successful preparation of this construct provided the key building block required for subsequent peptide conjugation strategies.

To enable alternative conjugation strategy based on disulfide exchange, a second PNA analogue bearing an N‐terminal cysteine residue was synthesised using the same optimised SPPS protocol.

Despite the use of scavengers such as DODT and EDT, a minor product was observed after cleavage, indicating that a modification originated from the intrinsic reactivity of the Boc‐protected cysteine under the cleavage conditions. Nevertheless, the desired Cys‐PNA product was successfully isolated by RP‐HPLC and characterised by mass spectrometry (Figure S4).

To serve as a negative control, a scrambled PNA sequence with no predicted complementarity to miRNA‐221‐5p was synthesised under the same optimised SPPS conditions (Table 1, Entry 2). Unlike the anti‐miRNA sequence, the scrambled construct did not exhibit significant aggregation or deletion products during synthesis, thanks to the optimised conditions.

### 3.2 Synthesis of Fluorescently Labelled CPPs

To facilitate the intracellular delivery of PNA cargo, two CPPs were selected: the purely cationic Tat peptide [44, 45] and the amphipathic Penetratin peptide (PNT), in which the Met residue was replaced with norleucine to avoid methionine oxidation during synthesis and handling (Table 2) [46]. These peptides were synthesised using automated SPPS with double coupling cycle to maximise coupling efficiency and minimise deletion sequences.

**TABLE 2:** CPPs sequences synthesised.

Compound	Sequence
*CF*‐Tat‐Ahx‐Aza‐NH_2_	*CF*‐GRKKRRQRRR‐Ahx‐Aza‐NH_2_
*CF*‐Tat‐Ahx‐Cys (TNP)*‐NH_2_	*CF*‐GRKKRRQRRR‐Ahx‐Cys (TNP)‐NH_2_
*CF*‐PNT‐Ahx‐Aza‐NH_2_	*CF*‐RQIKIWFQNRRNleKWKK‐Ahx‐Aza‐NH_2_
*CF*‐PNT‐Ahx‐Cys (TNP)*‐NH_2_	*CF*‐RQIKIWFQNRRNleKWKK‐Ahx‐Cys (TNP)‐NH_2_

*Note:* CF: carboxyfluorescein; Ahx: 6‐aminohexanoic acid, Cys (TNP)*: Cys (ThioNitroPyridine).

To enable fluorescent‐based monitoring of cellular uptake, the N‐terminus of each peptide was labelled with carboxyfluorescein (CF) via HATU/DIPEA activation.

Additionally, an azide group was introduced at the C‐terminus, using (S)‐2‐(9‐fluorenylmethyloxycarbonylamino)‐3‐azidopropanoic acid (Aza), to enable subsequent conjugation with alkyne‐functionalised PNA via click chemistry. The azide‐bearing CPPs were synthesised on Rink amide resin because the azido functionalities can undergo side reactions when anchored to supports such as 2‐chlorotrityl chloride resin. Specifically, when the azide is bound as a trityl ester, exposure to Fmoc deprotection solution can result in elimination, forming dehydroalanine. This is then subject to nucleophilic attack, leading to peptide degradation and the formation of by‐products. Consequently, Rink amide resin was selected as a more suitable support for preparing azide‐functionalised CPPs [47].

An optimisation of the synthetic route for cysteine‐containing CPP derivatives, intended for disulfide‐mediated conjugation, was performed synthesising short arginine‐rich sequences on Rink‐amide resin. LC–MS analysis revealed multiple fragmentation peaks and low‐mass by‐products, indicating incomplete coupling and partial side‐chain degradation during synthesis or cleavage. These issues were observed consistently across different spacer variants, suggesting that the instability was primarily associated with the solid support rather than the peptide sequence itself (Figure S9).

To overcome this, the synthesis was transferred to 2‐chlorotrityl chloride (2‐CTC) resin, which offered milder cleavage conditions and greatly enhanced the stability of the cysteine‐containing peptides. This modification was essential for reliably preparing CPP derivatives for subsequent disulfide‐based conjugation with PNA cargo (Table 2).

### 3.3 CuAAC‐Mediated Formation of CPP‐PNA Conjugates

Alkyne‐functionalised PNA was conjugated to azide‐modified CPPs via CuAAC click chemistry. This reaction was chosen for its high efficiency, chemoselectivity and compatibility with aqueous conditions [48, 49]. The conjugation reactions were performed in an H_2_O/*t*‐BuOH solvent mixture, in the presence of CuSO_4_ and sodium ascorbate to generate the catalytically active Cu(I) species in situ (Figure 2A).

**FIGURE 2:**
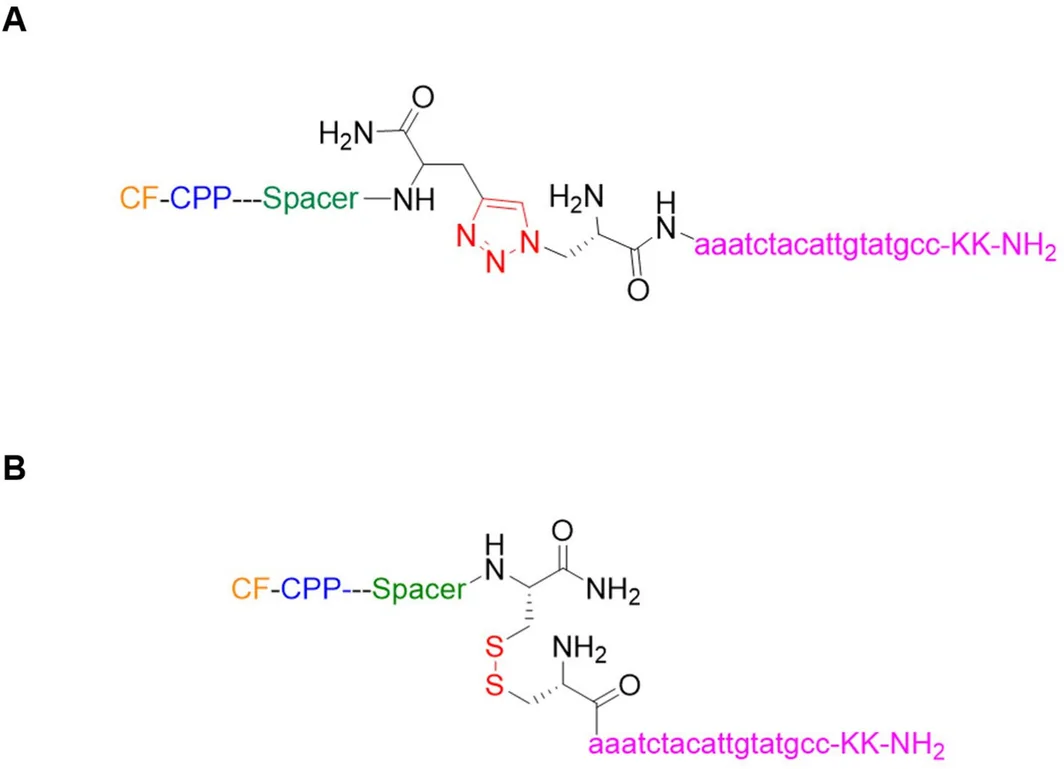
Schematic representation of the conjugates obtained through triazole linker (A) and disulfide bond (B).

The resulting triazole linker is highly chemically stable and resistant to enzymatic degradation, ensuring that the CPP remains covalently attached to the PNA during cellular uptake. This stability is a key advantage of the CuACC strategy when it comes to creating robust CPP‐PNA constructs for use in biological applications.

### 3.4 Disulfide‐Linked CPP‐PNA Conjugates

In addition to the permanent triazole linkage generated by click chemistry, a second conjugation strategy based on the formation of reversible disulfide bonds was explored. Disulfide linkers are appealing due to their ability to be cleaved in a reducing environment, such as that found in the cytosol. This could potentially enable the intracellular release of the PNA cargo.

To favour selective heteroconjugation and prevent the formation of unwanted homodimers, the cysteine residue of the CPP was first activated using 2,2‐dithiopyridine (DTNP), generating a mixed disulfide intermediate (Table 2). This activated peptide was then reacted with PNA bearing a cysteine residue via thiol‐disulfide exchange under mild conditions (Figure 2B) [50].

Compared to CuAAC coupling, the disulfide exchange strategy was found to be more sensitive to reaction conditions and generally produced lower overall yields. However, the reversible nature of the disulfide linkage offers an advantage in biological systems where intracellular reduction facilitates the release of active antisense PNA, avoiding potential interference of the CPP with the PNA intracellular target.

### 3.5 Structural Characterization and miRNA‐PNA Interactions by Circular Dichroism

CD spectroscopy was used to study the conformational behaviour of miRNA‐221‐5p and evaluate its interactions with complementary anti‐miRNA‐221‐5p PNA and a scrambled control sequence. For these experiments, anti‐miRNA PNA and the scrambled control PNA with free amine at the N‐terminus (Table 1) were used.

The CD spectrum of miRNA‐221‐5p alone displayed the typical features associated with a right‐handed RNA helical conformation: a positive band centred around 266 nm and a negative band near 205 nm. These spectral characteristics are consistent with the expected folding behaviour of RNA oligonucleotides in aqueous buffer conditions.

Upon the addition of the complementary anti‐miRNA‐221‐5p PNA, significant spectral changes were observed. In particular, the intensity of the positive band at about 260 nm increased markedly, accompanied by a blue shift of the maximum and enhanced ellipticity around 220 nm (Figure 3) [51]. Notably, the experimental CD spectrum of the mixture diverged significantly from the sum of the individual spectra of the two strands. This deviation suggests that the observed signal originates from a newly formed molecular structure, rather than being the result of the simple addition of the individual components. Taken together, these features are consistent with the formation of a well‐defined PNA‐RNA heteroduplex characterised by enhanced base stacking and increased helicity.

**FIGURE 3:**
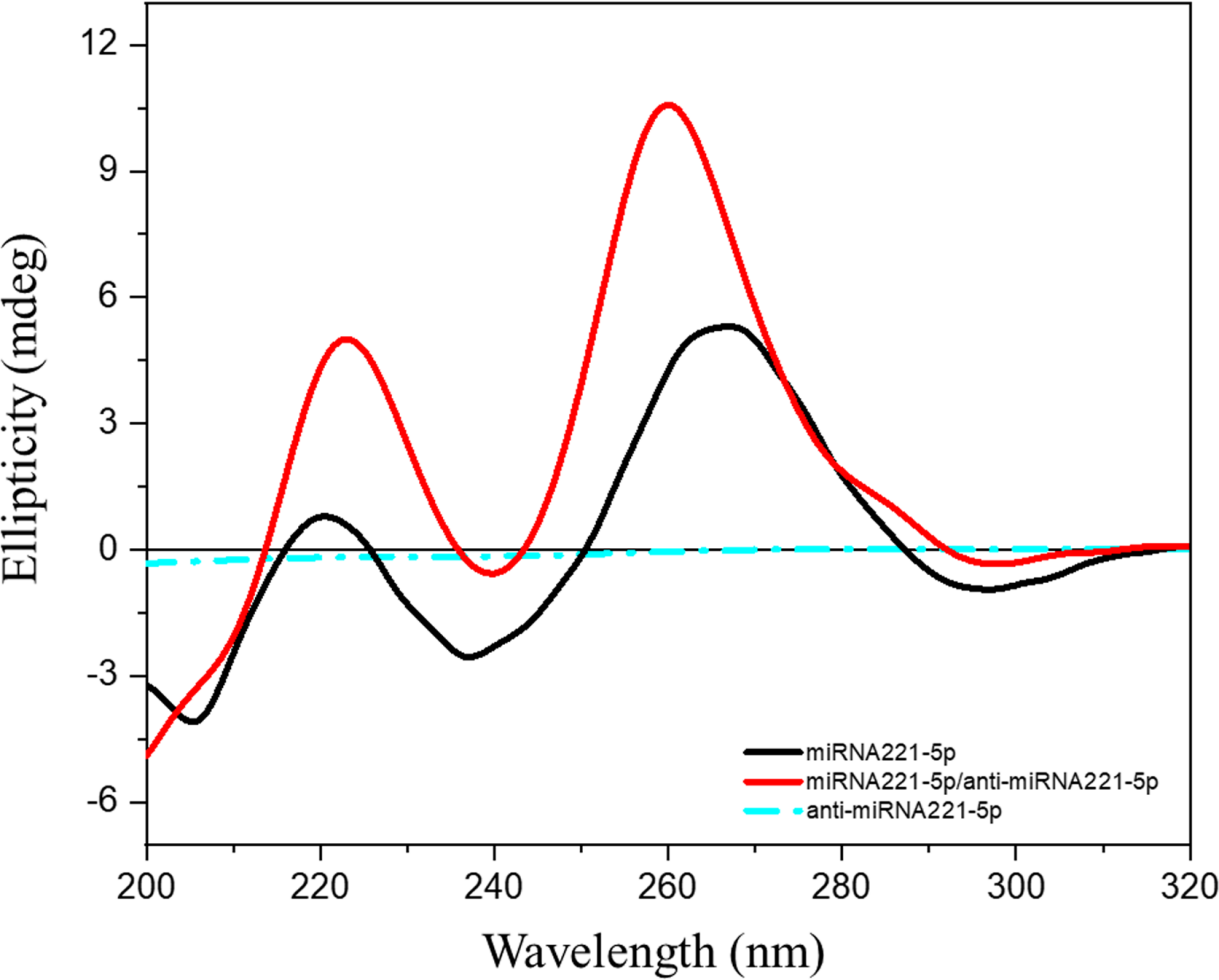
CD spectra of 2.5 μM of miRNA‐221‐5p (in black), miRNA‐221‐5p/anti‐miRNA‐221‐5p PNA duplex (in red) and anti‐miRNA‐221‐5p PNA (dashed line) in 10 mM of phosphate buffer at pH = 7.4, in the presence of 10 mM of NaCl.

By contrast, incubating miRNA‐221‐5p with the scrambled PNA sequence produced minimal changes to the spectrum (Figure 4). The resulting spectrum closely overlapped with that of the free miRNA and with the calculated sum of the individual spectra of the two strands. The absence of significant wavelength shifts or ellipticity changes suggests that no stable duplex structure formed under these conditions. These observations demonstrate that the structural changes observed in the miRNA‐221‐5p/anti‐miRNA‐221‐5p PNA system arise from specific Watson–Crick base pairing, rather than from nonspecific electrostatic or stacking interactions between the nucleic components.

**FIGURE 4:**
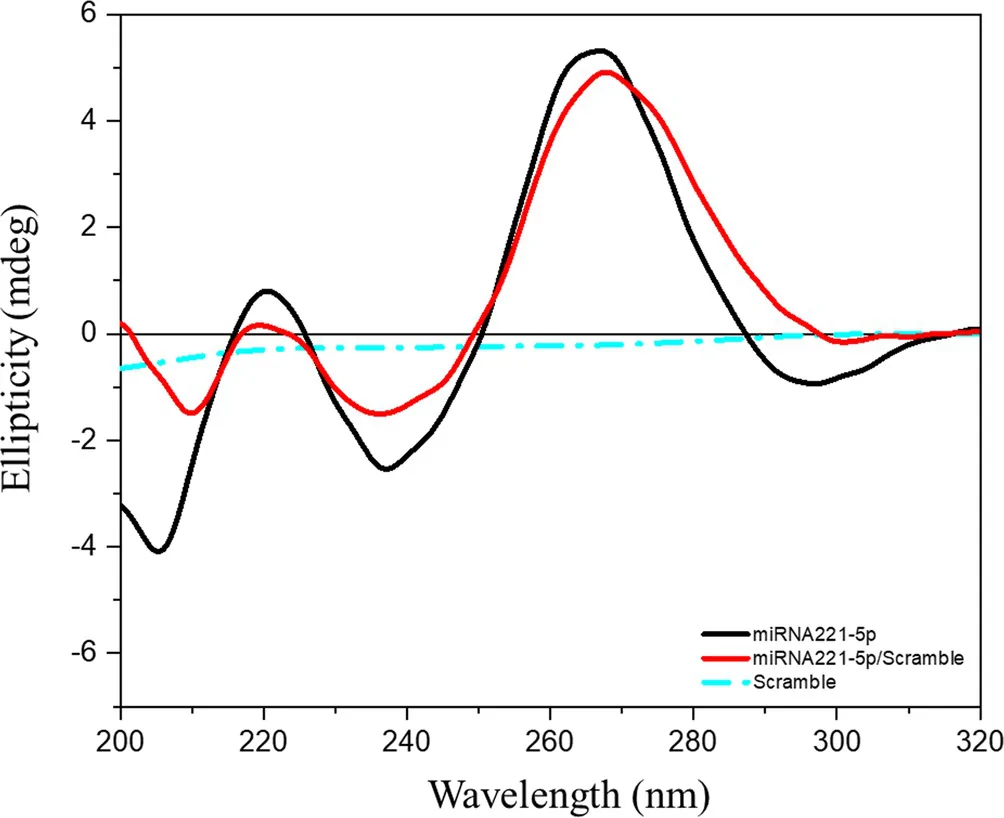
CD spectra of 2.5 μM of miRNA‐221‐5p (in black), miRNA‐221‐5p/scrambled PNA complex (in red) and scrambled PNA in 10 mM of phosphate buffer at pH = 7.4, in the presence of 10 mM of NaCl.

Overall, the CD analysis provides compelling evidence that the designed anti‐miRNA‐221‐5p PNA specifically interacts with the target miRNA sequence, inducing the formation of a more ordered and stable duplex structure. The lack of comparable changes in the scrambled control sample provides further support for the sequence‐selective nature of the PNA‐RNA interaction.

### 3.6 Thermal Stability of miRNA‐PNA Duplexes

Thermal denaturation experiments were conducted to examine the strength and specificity of the interaction between miRNA‐221‐5p and the designed PNA constructs (Table 3).

**TABLE 3:** Summary of the T_m_ found.

Sample	T_m_ (°C)
miRNA‐221‐5p	53 ± 2.8
miRNA‐221‐5p/anti‐miRNA‐221‐5p PNA	72 ± 3.7
miRNA‐221‐p/scrambled	62 ± 2.5

The free miRNA‐221‐5p strand displayed a melting temperature of 53°C (Table 3, Entry 1). A substantial increase in thermal stability was observed when miRNA‐221‐5p was hybridised with the complementary anti‐miRNA‐221‐5p PNA (Table 3, Entry 2, and Figure 5).

**FIGURE 5:**
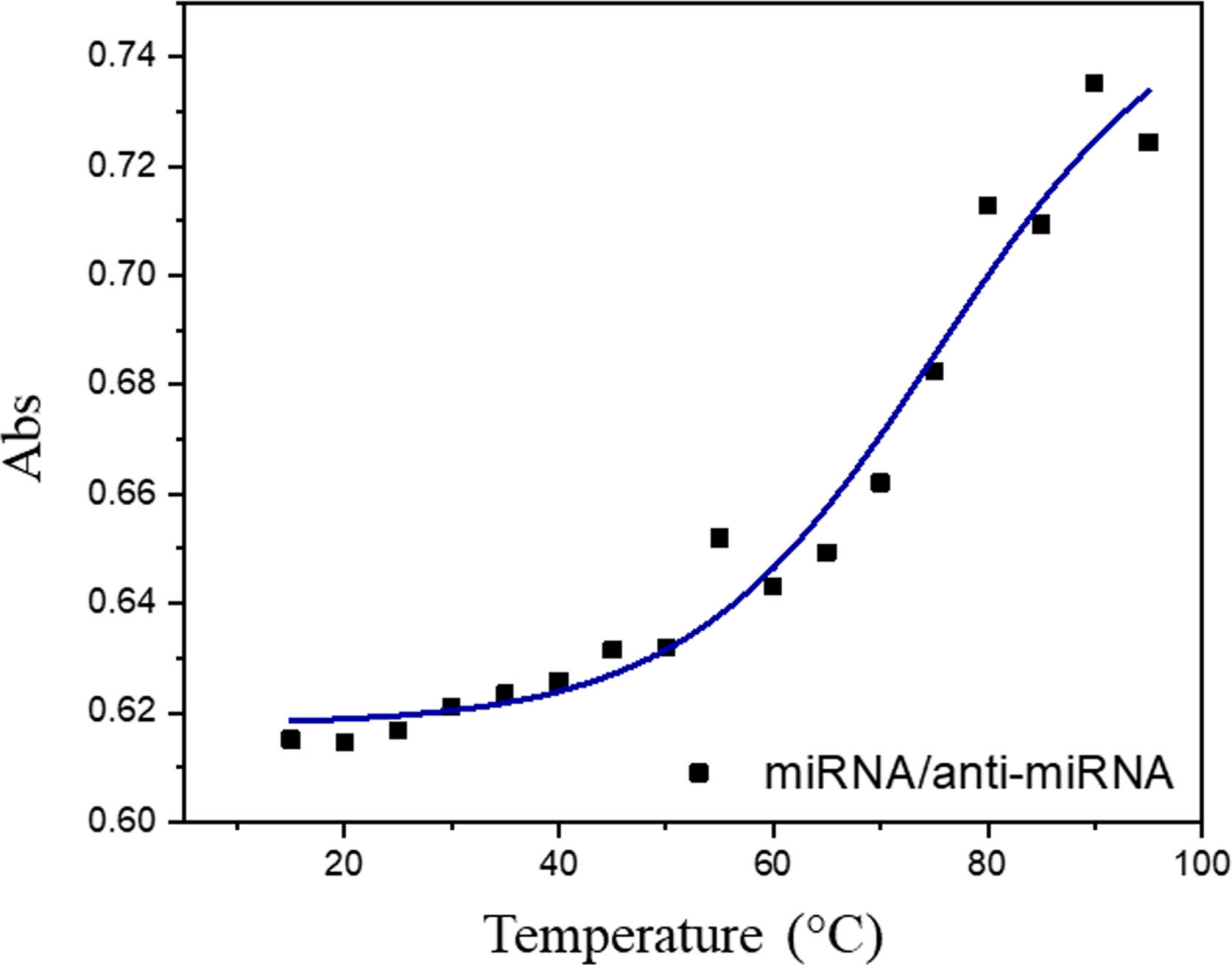
UV‐melting curves of miRNA 221‐5p/anti‐miRNA‐221‐5p duplex at 2.5‐μM concentration in 10 mM of NaCl, 10 mM of phosphate buffer, pH = 7.4. Detection at 260 nm.

The resulting heteroduplex had a melting temperature of 72°C, which is nearly 20°C higher than that of the free RNA strand. This pronounced stabilization reflects the formation of a highly stable PNA‐RNA duplex, which is consistent with the well‐known high binding affinity of PNAs for complementary nucleic acid targets. This enhanced stability is primarily due to the neutral backbone of the PNA, which eliminates the electrostatic repulsion that is usually present in nucleic acid duplexes, thereby promoting stronger base pairing and stacking interactions.

In contrast, the duplex formed between miRNA‐221‐5p and the scrambled PNA sequence had a melting temperature of only 62°C, which was barely higher than that of the miRNA alone (Table 3, Entry 3). This limited increase suggests that the scrambled sequence does not form a fully complementary duplex with the target RNA. The moderate increase in melting temperature observed for the scrambled PNA is most likely due to the presence of a short region containing five consecutive complementary nucleobases that can form local Watson–Crick interactions with miRNA‐221‐5p. While these interactions offer some thermal stabilisation, they are insufficient to generate the continuous, well‐defined PNA‐RNA heteroduplex necessary for the characteristic CD spectral changes seen with the fully complementary anti‐mRNA‐221‐5p PNA.

### 3.7 Biological Evaluation of CPP‐PNA Conjugates Targeting miRNA‐221‐5p

After synthesising and physico‐chemically characterising the PNA conjugates, we performed preliminary biological assays to evaluate their cytocompatibility and potential antiproliferative activity.

#### 3.7.1 Antiproliferative Activity of CPP‐PNA Conjugates

The biological activity of CPP‐PNA conjugates was evaluated in A549 human lung cancer cells. To investigate the cellular effects of the conjugates, the screening experiments were conducted at 5‐μM concentration of conjugates.

Cell viability for treated samples relative to untreated controls was assessed after 1, 24 and 48 h using the CCK‐8 assay (Figure 6) [52]. The primary objective was to distinguish between two possible outcomes: genuine antiproliferative activity resulting from the inhibition of miRNA‐221‐5p at a specific sequence detectable after long incubation (24 and 48 h) and nonspecific cytotoxicity (e.g., membrane perturbation) detectable after short incubation (1 h).

**FIGURE 6:**
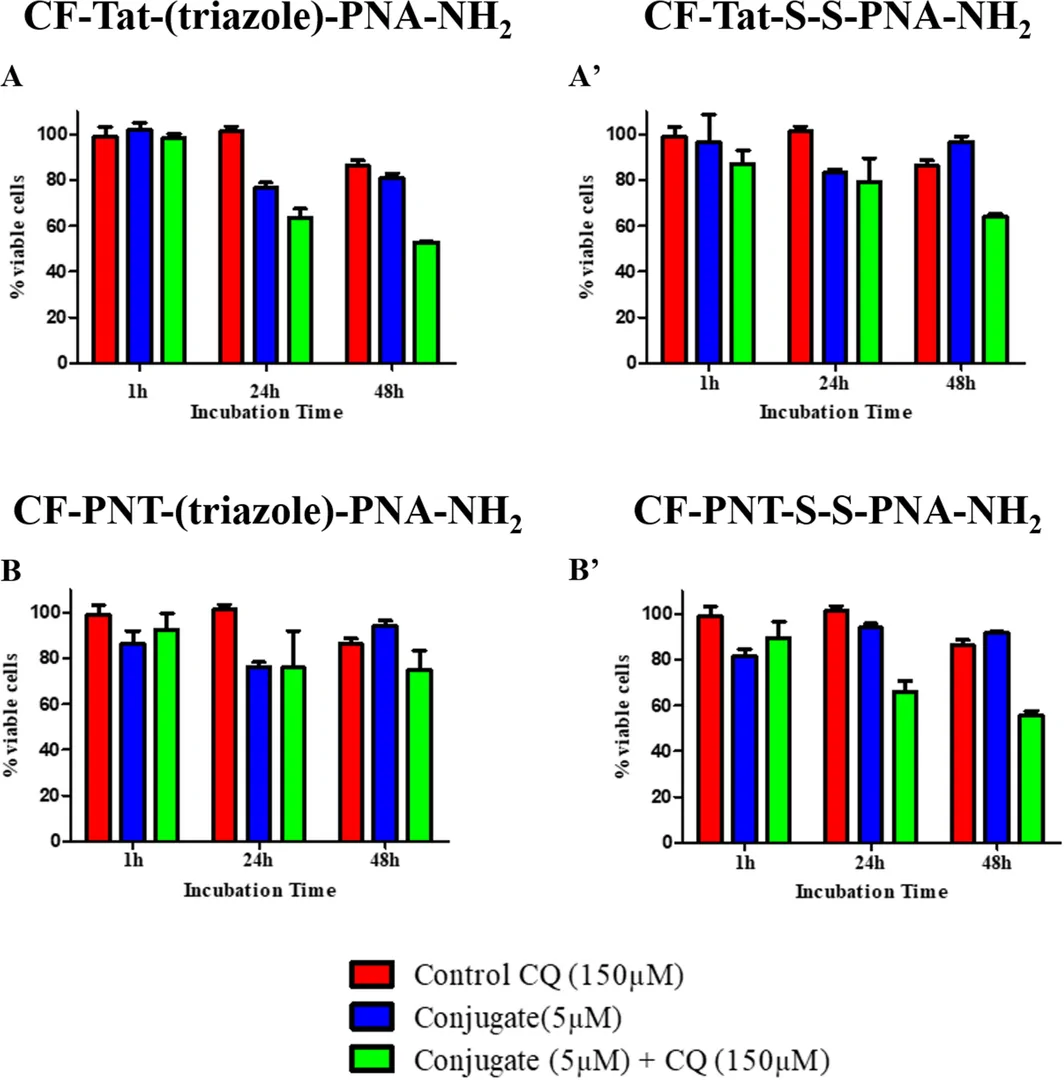
Cell proliferation assays. CF‐Tat‐(triazole)‐PNA (A), CF‐Tat‐S‐S‐PNA (A') and. CF‐PNT‐(triazole)‐PNA (B), CF‐PNT‐S‐S‐PNA (B′), were tested on A549 cells at a final concentration of 5 μM, either alone or in combination with chloroquine (CQ) 150 μM. Cell viability was evaluated after 1, 24 and 48 h using the CCK‐8 assay. The percentage of viable cells is expressed relative to the corresponding control, consisting of cells incubated for the same duration under identical conditions (complete culture medium and serum) but without peptide or additives.

Importantly, after 1 h of exposure with the conjugates, cell viability remained above 80% for all conjugates indicating that they do not cause acute membrane toxicity. This suggests that any subsequent changes in cell proliferation arise from intracellular mechanisms rather than nonspecific membrane permeabilization. However, none of the CPP‐PNA conjugates induced a significant antiproliferative effect over 48 h, implying that the antisense PNAs failed to efficiently reach their intracellular targets, potentially due to endosomal entrapment. To enhance cytosolic delivery, the conjugates were co‐incubated with chloroquine (150 μM), a weak base known to destabilise endosomal membranes (Figure 6) [53]. Additionally, the effect of calcium ions (5 mM) was tested (Figure S19), as previous studies demonstrated their ability to improve the antisense activity of PNAs conjugated to cationic CPPs by improving conjugate interactions with the cell membrane and also facilitating endosomal escape [54].

Calcium ions (5 mM) produced intrinsic toxicity over time or interfered with cell proliferation yet failed to enhance the biological activity of the CPP‐PNA constructs (Figure S19). In contrast, chloroquine (150 μM) alone showed no cytotoxicity or impact on proliferation over 48 h. Interestingly, its co‐incubation with the PNA conjugates resulted in a reduction of the percentage of viable cells after 24 and 48 h (Figure 6), consistent with promoted endosomal escape of the conjugates.

A comparison of the different CPP‐PNA conjugates revealed clear differences depending on both the CPP and the chemistry of the linker used for conjugation. The PNA linked to the Tat CPP via a triazole linker and to Penetratin via a disulfide bond gave the best result with approximately 50% reduction in tumor cell number after 48 h in both cases. This was followed by the disulfide‐linked Tat‐PNA conjugate (35% decrease in 48 h). In contrast, the PNT‐triazole‐PNA conjugate failed to produce any significant biological activity even when chloroquine was present.

Disulfide linkages are appealing as they can be cleaved in the reducing cytosolic environment, which contains millimolar concentration of glutathione [55]. However, they are susceptible to cell surface thiol‐disulfide exchange reactions [56]. These reactions can form disulfide‐linked species between cysteine‐containing membrane proteins and the conjugate moieties, leading to premature cleavage of the conjugates in the extracellular media. The cross‐linked species may either remain trapped at the membrane or be internalised by endocytosis.

Our results suggest that the occurrence and outcome of cell surface thiol/disulfide reactions depend on the CPP sequence, as previously observed for disulfide peptide conjugates [57]. These findings emphasise the importance of testing the efficiency of various CPPs in combination with linkers of different nature. With the Penetratin CPP, the disulfide linkage was more favourable, possibly due to enhanced endocytosis of the PNA via a mechanism involving cell surface thiols. In contrast, when using the Tat CPP, the triazole linkage—improving the robustness and intracellular persistence of the CPP‐PNA construct—appeared to favour the functional delivery of the antisense cargo.

#### 3.7.2 Quantification of CPP‐PNA Efficiency of Cellular Uptake

To quantitatively assess the intracellular uptake of the CPP‐PNA conjugates, adherent A549 cells were incubated for 1 h at 37°C with the fluorescent conjugates (5 μM in DMEM) before being analysed using the fluorometry‐based protocol described by Illien et al. [57]. This protocol combines the enzymatic removal of membrane‐bound species with the quantitative fluorescence measurement of cell lysates. This approach selectively quantifies the intracellular fraction of the conjugates, eliminating the contribution of surface‐bound material. Crucially, the method provides the total amount of internalised fluorescent species (including intact and degraded/reduced conjugates) as fluorescence is measured following full cell lysis, irrespective of their intracellular localisation, including both cytosol and vesicular/endosomal compartments.

As shown in Figure 7, all four CPP‐PNA conjugates were successfully internalised by A549 cells, albeit with markedly different efficiencies depending on the CPP sequence and conjugation strategy employed. Among the tested constructs, CF‐PNT‐(triazole)‐PNA‐NH_2_ displayed the highest cellular uptake efficiency, followed by CF‐Tat‐(triazole)‐PNA‐NH_2_, whereas both disulfide‐linked conjugates gave substantially lower intracellular levels. These results demonstrate that both Tat and PNT efficiently promote the cellular uptake of the PNA cargo and further indicate that linker chemistry significantly influences the overall efficiency of intracellular delivery.

**FIGURE 7:**
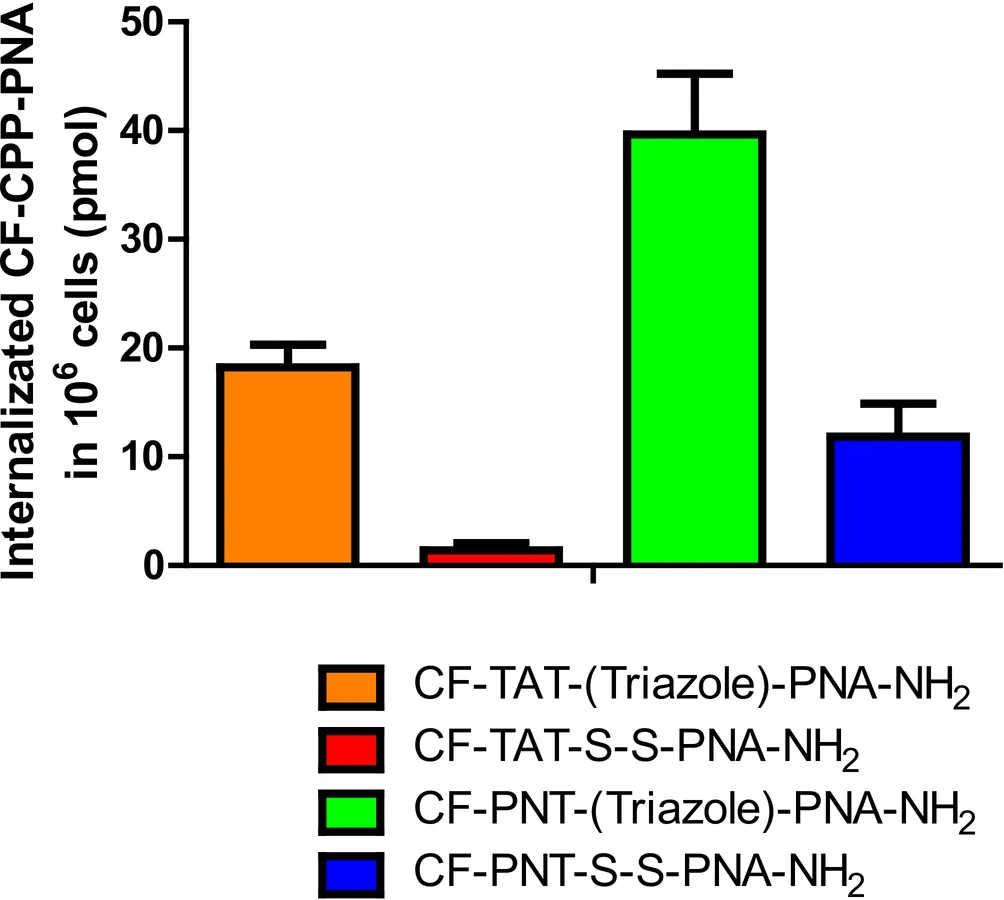
Quantification by fluorometry of internalised CF‐CPP‐PNA in A549 cells. Adherent cells (10^6^) were incubated for 1 h at 37°C with the different CF‐CPP‐PNA conjugates (5 μM in DMEM).

Notably, the quantitative uptake values did not fully correlate with the antiproliferative activities observed in the biological assays. This discrepancy is not unexpected and likely reflects the fact that cellular uptake does not necessarily correspond to bioavailability within the cell. The fluorometric method quantifies the overall amount of conjugate derivative present inside the cell. It does not discriminate between the derivatives present in intracellular organelles and those successfully released into the cytosol where the PNA can hybridise with its target miRNA and exert its biological activity. Endosomal escape is widely recognised as one of the main limitations of CPP‐mediated nucleic acid delivery [36, 53], as also suggested by the fact that chloroquine was required in our antiproliferative experiments to observe a biological response. Therefore, differences in endosomal release efficiency between conjugates may contribute to the lack of a direct relationship between the measured amounts of internalised species and the antiproliferative activities. The nature of the linker between the CPP and PNA may also explain the observed lack of correlation, as the CPP, when conjugated via a stable triazole linker, may perturb PNA binding to its target. Finally, the amphiphilic nature of the CPP‐PNA conjugates may lead to self‐association or aggregation at the cell surface or inside cells. Such aggregation phenomena could influence the internalisation efficiency and the effective concentration of the freely diffusible PNA available for engaging with the intracellular target, providing an additional explanation for the observed differences between cellular uptake and biological response.

Overall, these findings suggest that cellular internalisation alone cannot predict the biological activity of CPP‐PNA conjugates. This highlights the fact that efficient intracellular delivery is the result of a combination of cellular uptake, intracellular trafficking, preservation of the structural integrity of the conjugates and capacity to reach and interact with the intracellular target. Further studies specifically quantifying cytosolic delivery would be valuable in order to better correlate intracellular availability with biological efficacy.

## 4 Conclusion

This study presents an integrated chemical, biophysical and biological approach to developing PNA conjugates functionalised with CPPs for use as delivery systems in the antisense targeting of the oncogenic miRNA‐221‐5p. The synthetic strategy combines SPPS with two complementary conjugation methodologies: CuAAC click chemistry and disulfide‐mediated coupling. This allows the preparation of structurally diverse CPP‐PNA conjugates. While both approaches were effective in generating PNA constructs, the triazole linkage formed via CuAAC offered superior chemical robustness, whereas disulfide linkages provided the potential advantage of intracellular cargo release.

Spectroscopic studies confirmed that the designed PNA sequence could selectively recognise its target microRNA. Circular dichroism and UV‐melting analyses demonstrated the formation of a stable, sequence‐specific PNA‐RNA heteroduplex with miRNA‐221‐5p, characterised by increased helicity and a significantly higher melting temperature than the free RNA strand. Control experiments using a scrambled PNA sequence showed no comparable structural changes, confirming that the observed interactions arise from specific base pairing rather than nonspecific electrostatic effects.

Preliminary biological investigations provided insights into intracellular behaviour and activity of the CPP‐PNA conjugates. Cell proliferation assays revealed initial evidence of biological activity for two conjugates: CF‐Tat‐(triazole)‐PNA‐NH_2_ and CF‐PNT‐S‐S‐PNA‐NH_2_. These produced, in presence of chloroquine, a measurable (50%) reduction in A549 lung cancer cell proliferation after 48 h at micromolar concentrations. Importantly, these effects were not associated with acute cytotoxicity or membrane disruption, which supports the hypothesis that they arise from the intracellular engagement of the target miRNA rather than nonspecific peptide toxicity. While the observed effects are modest, they suggest that some of the conjugates successfully reached the cytosol and modulated proliferative signalling pathways.

Overall, this work establishes a methodological foundation for the synthesis, characterisation, quantitative cellular uptake and preliminary biological evaluation of CPP‐PNA conjugates that target oncogenic miRNAs. The internalisation data confirm that both Tat‐ and PNT‐based CPPs mediate the cellular uptake of PNA cargoes. No direct correlation was observed between the amounts of internalised species and the antiproliferative activities highlighting the fact that intracellular activity also depends on subsequent events, including endosomal escape and intracellular trafficking. These events determine the fraction of the conjugate that becomes biologically available to hybridise with the target miRNA. Additionally, the results emphasise the critical role of the linker in determining the biological performance of specific CPP constructs. Biological activity was detected only when conjugates were incubated with cells in the presence of chloroquine, indicating that while Tat and Penetratin CPPs mediated PNA cellular uptake via endocytosis, they were unable to promote endosomal escape. Future studies will focus on exploring alternative CPP architectures, such as hybrid vectors incorporating a Trp‐rich endosomal escape domain [58] or oligo‐His peptides acting as proton sponge [59], to enhance cytosolic delivery.

Taken together, these findings provide valuable insights into designing peptide‐based delivery systems for nucleic acid therapeutics, supporting the continued development of CPP‐PNA conjugates as potential tools for targeting and modulating oncogenic microRNA pathways.

## Conflicts of Interest

The authors declare no conflicts of interest.

## Supporting information


**Figure S1:** Chemical structure of PNA anti‐miRNA‐221‐5p together with HRMS spectra. Molecular formula for C_206_H_267_N_105_O_56_. Calculated molecular weight: 5107.13 Da. Experimental m/z: [M + 3H]^3+^: 1703.3770 (A); [M + 4H]^4+^: 1277.8044 (B); [M + 5H]^5+^: 1022.4279 (C); [M + 6H]^6+^: 852.2010 (D); [M + 7H]^7+^: 730.5889 (E).
**Figure S2:** Chemical structure of the Alkyne‐PNA‐KK‐NH_2_ PNA together with the ESI‐MS spectrum. Molecular formula: C_212_H_273_N_105_O_57_, Calculated molecular weight: 5204.7 Da; Experimental m/z: [M + 2H]^2+^: 2603.2, [M + 3H]^3+^: 1735.8, [M + 4H]^4+^: 1302.1.
**Figure S3:** Chemical structure of the Alkyne‐PNA‐KK‐NH_2_ PNA together with the HRMS spectra (A) Molecular formula: C_212_H_273_N_105_O_57_, Calculated molecular weight: 5204.7 Da; Experimental m/z: [M + 3H]^3^: 1735.3940 (B); [M + 4H]^4+^: 1041.6449 (C).
**Figure S4:** Chemical structure of the Cys‐PNA‐KK‐NH_2_ PNA together with the ESI‐MS spectrum. Molecular formula: C_209_H_272_N_106_O_57_S, [M + H]^1+^_Calculated_: 5212.8 Da; Experimental mass: [M + 2H]^2+^: 2606.7, [M + 3H]^3+^: 1738.1, [M + 4H]^4+^: 1303.8.
**Figure S5:** Chemical structure of the H‐Scramble‐KK‐NH_2_ PNA together with the ESI‐MS spectrum. Molecular formula: C_206_H_267_N_105_O_56_, [M + H]^1+^_Calculated_: 5108.8. Experimental mass: [M + 2H]^2+^: 2554.8, [M + 3H]^3+^: 1703.5, [M + 4H]^4+^: 1277.9.
**Figure S6:** Chemical structure of the H‐Scramble‐KK‐NH2 PNA together with the HRMS spectra. Molecular formula for C_206_H_267_N_105_O_56_. Calculated molecular weight: 5107.13 Da. Experimental m/z: [M + 3H]^3+^: 1703.3770 (A); [M + 4H]^4+^: 1277.7780 (B); [M + 5H]^5+^: 1022.7249 (C); [M + 6H]^6+^: 852.2010 (D); [M + 7H]^7+^: 730.5889 (E).
**Figure S7:** Chemical structure of the CF‐Tat‐N_3_‐NH_2_ together with the ESI‐MS spectrum. Molecular formula: C_85_H_135_N_37_O_19_, [M + H]^1+^_Calculated_: 1979.1; Experimental mass: [M + H] ^+^: 1977.9, [M + 2H]^2+^: 989.8, [M + 3H]^3+^: 660.3.
**Figure S8:** Chemical structure of CF‐PNT‐Ahx‐N_3_‐NH_2_ together with the ESI‐MS spectrum. Molecular formula: C_135_H_195_N_39_O_28_, [M + H]^1+^_Calculated_: 2813.3; Experimental mass: [M + H]^+^: 2811.6, [M + 2H]^2+^: 1405.7, [M + 3H]^3+^: 937.6.
**Figure S9:** Analytical HPLC chromatogram of the Fmoc‐RRR‐Ahx‐C‐NH_2_ (A) Fmoc‐RRR‐βAla‐C‐NH_2_ (B) Fmoc‐RRR‐C‐NH_2_ (C) crude, after the cleavage, recorded using a Kinetex C18 column, 1 mL/min flow rate, with detection at 220 nm. Elution was initially performed at 5% of solvent B, followed by a linear gradient from 5% to 95% over 30 min. The product peak, if present, is indicated by the arrow.
**Figure S10:** Chemical structure of the CF‐Tat‐Ahx‐Cys (TNP)‐OH together with the ESI‐MS spectrum. Molecular formula: C_90_H_137_N_35_O_22_S_2_, [M + H]^1+^_Calculated_: 2125.3; Experimental mass: [M + H]^+^: 2124.9, [M + 2H]^2+^: 1062.9, [M + 3H]^3+^: 709.0, [M + H]^+#^: 1968.9 represents the peptide experimental mass after the loss of the activation on the Cys residue.
**Figure S11:** Chemical structure of CF‐PNT‐Cys (TNP)‐OH together with the ESI‐MS spectrum. Molecular formula: C_140_H_198_N_38_O_30_S_2_, [M + H]^1+^_Calculated_: 2958.2; Experimental mass: [M + H]^+^: 2957.9, [M + 2H]^2+^: 1478.8, [M + 3H]^3+^: 986.3.
**Figure S12:** Chemical structure of CF‐Tat (triazole)PNA‐NH_2_ together with ESI‐MS spectrum. Molecular formula: C_296_H_406_N_142_O_76_, [M + H]^1+^_Calculated_: 7181.5; Experimental mass: [M + 3H]^3+^: 2396.1, [M + 4H]^4+^: 1795.8.
**Figure S13:** Chemical structure of CF‐PNT (triazole)PNA‐NH_2_ together with the ESI‐MS spectrum. Molecular formula: C_346_H_467_N_145_O_76_, [M + H]^1+^_Calculated_: 8020.0; Experimental mass: [M + 3H]^3+^: 2671.7, [M + 4H]^4+^: 2004.0, [M + 5H]^5+^: 1603.3.
**Figure S14:** Chemical structure of CF‐Tat‐S‐S‐PNA‐NH_2_ together with the ESI‐MS spectrum. Molecular formula: C_294_H_406_N_140_O_76_S_2_, [M + H]^1+^_Calculated_: 7181.7; Experimental mass: [M + 3H]^3+^: 2396.1, [M + 4H]^4+^: 1795.8, [M + 5H]^5+^: 1437.0.
**Figure S15:** Chemical structure of CF‐PNT‐S‐S‐PNA‐NH_2_ together with the ESI‐MS spectrum. Molecular formula: C_344_H_467_N_143_O_84_S_2_, [M + H]^1+^_Calculated_: 8014.4; Experimental mass: [M + 3H]^3+^: 2671.7, [M + 4H]^4+^: 2004.1, [M + 5H]^5+^: 1603.3.
**Figure S16:** UV‐melting curves at 260 nm of miRNA 221‐5p at 2.5‐μM concentration in 10 mM of NaCl, 10 mM of phosphate buffer pH = 7.4.
**Figure S17:** UV‐melting curves at 260 nm of miRNA 221‐5p/scramble duplex at 2.5‐μM concentration in 10 mM of NaCl, 10 mM of phosphate buffer pH = 7.4.
**Figure S18:** Cell proliferation assays. CF‐Tat‐(triazole)‐PNA (A), CF‐Tat‐S‐S‐PNA (A') and CF‐PNT‐(triazole)‐PNA (B), CF‐PNT‐S‐S‐PNA (B′) were tested on A549 cells at a final concentration of 5 μM, either alone or in combination with calcium ions (Ca^2+^) 5 mM. Cell viability was evaluated after 1, 24 and 48 h using the CCK‐8 assay. The percentage of viable cells is expressed relative to the corresponding control, consisting of cells incubated for the same duration under identical conditions (complete culture medium and serum) but without peptide or additives.

## Data Availability

The data that support the findings of this study are available on request from the corresponding author. The data are not publicly available due to privacy or ethical restrictions.
