# Supplementary material for: Design, Synthesis and Biophysical Characterisation of CPP‐PNA Conjugates for Targeting Oncogenic microRNA‐221
Source: J Pept Sci. 2026 Oct 2;32(11):e70129. doi: 10.1002/psc.70129 (PMC13631804; doi:10.1002/psc.70129)
Supplement: Supplementary file 1 [file PSC-32-e70129-s001.docx]

**Design, Synthesis, and Biophysical Characterization CPP-PNA Conjugates for Targeting Oncogenic microRNA-221**

Martina Rotondo,^1,3^ Françoise Illien,^2^ Fabienne Burlina,^2^ and Barbara Biondi^3^

*^1^Università Degli Studi di Napoli Federico II, Naples, Italy;*

*^2^Sorbonne Université, École Normale Supérieure, Université PSL, CNRS, Chimie Physique et Chimie du Vivant, CPCV, F-75005 Paris, France*

*^3^Institute of Biomolecular Chemistry of CNR, Padova Unit, Padova, Italy.*

- **Figure 1S.** HRMS Spectrometry of anti-miRNA-221-5p.
- **Figure 2S.** ESI-MS spectrum of the Alkyne-PNA-KK-NH_2_ PNA.
- **Figure 3S.** HRMS Spectrometry of anti-miRNA-221-5p.
- **Figure 4S.** ESI-MS spectrum of the Cys-PNA-KK-NH_2_ PNA.
- **Figure 5S.** ESI-MS spectrum of the H-Scramble-KK-NH_2_ PNA.
- **Figure 6S*.*** HRMS Spectrometry of H-Scramble-KK-NH2 PNA.
- **Figure 7S.** ESI-MS spectrum of CF-Tat-N_3_-NH_2_
- **Figure 8S.** ESI-MS spectrum of CF-PNT-Ahx-N_3_-NH_2_.
- **Figure 9S.** Analytical HPLC chromatogram of the Fmoc-RRR-Ahx-C-NH_2_ (**A**) Fmoc-RRR-βAla-C-NH_2_ (**B**) Fmoc-RRR-C-NH_2_ (**C**) crude
- **Figure 10S.** ESI-MS spectrum of CF-Tat-Ahx-Cys(TNP)-OH.
- **Figure 11S.** ESI-MS spectrum of CF-PNT-Cys(TNP)-OH.
- **Figure 12S.** ESI-MS spectrum of CF-Tat(triazole)PNA-NH_2_.
- **Figure 13S.** ESI-MS spectrum of CF-PNT(triazole)PNA-NH_2_.
- **Figure 14S.** ESI-MS spectrum of CF-Tat-S-S-PNA-NH_2_.
- **Figure 15S**. ESI-MS spectrum of CF-PNT-S-S-PNA-NH_2_.
- **Figure 16S.** UV-melting curves at 260 nm of miRNA 221-5p.
- **Figure 17S.** UV-melting curves at 260 nm of miRNA 221-5p/scramble duplex.
- **Figure 18S.** Cell proliferation assays of CF-Tat-(triazole)-PNA-NH_2_ (**A**) and CF-Tat-S-S-PNA-NH_2_ (**A’**) and CF-PNT-(triazole)-PNA-NH_2_ (**B**) and CF-PNT-S-S-PNA-NH_2_ (**B’**) at 5μM, either alone or in combination with calcium ions (Ca^2+^, 5 mM).

.

***Figure 1S.*** *Chemical structure of PNA anti-miRNA-221-5p together with HRMS spectra. Molecular formula for C_206_H_267_N_105_O_56_. Calculated molecular weight: 5107.13 Da. Experimental m/z: [M+3H]^3+^:1703.3770 (****A****); [M+4H]^4+^:1277.8044 (****B****); [M+5H]^5+^:1022.4279 (****C****); [M+6H]^6+^:852.2010 (****D****); [M+7H]^7+^:730.5889 (****E****).*

**Figure 2S.** Chemical structure of the Alkyne-PNA-KK-NH_2_ PNA together with the ESI-MS spectrum. Molecular formula: C_212_H_273_N_105_O_57_, Calculated molecular weight: 5204.7 Da; Experimental m/z: [M+2H]^2+^:2603.2, [M+3H]^3+^:1735.8, [M+4H]^4+^:1302.1.

***Figure 3S.*** *Chemical structure of the Alkyne-PNA-KK-NH_2_ PNA together with the HRMS spectra (****A****) Molecular formula: C_212_H_273_N_105_O_57_, Calculated molecular weight: 5204.7 Da; Experimental m/z: [M+3H]^3^: 1735.3940 (****B****); [M+4H]^4+^:1041.6449 (****C****).*

**Figure 4S.** Chemical structure of the Cys-PNA-KK-NH_2_ PNA together with the ESI-MS spectrum. Molecular formula: C_209_H_272_N_106_O_57_S, [M+H]^1+^_Calculated_: 5212.8 Da; Experimental mass: [M+2H]^2+^:2606.7, [M+3H]^3+^:1738.1, [M+4H]^4+^:1303.8.

**Figure 5S.** Chemical structure of the H-Scramble-KK-NH_2_ PNA together with the ESI-MS spectrum. Molecular formula: C_206_H_267_N_105_O_56_, [M+H]^1+^_Calculated_: 5108.8; Experimental mass: [M+2H]^2+^:2554.8, [M+3H]^3+^:1703.5, [M+4H]^4+^:1277.9.

***Figure 6S.*** *Chemical structure of the H-Scramble-KK-NH2 PNA together with the HRMS spectra. Molecular formula for C_206_H_267_N_105_O_56_. Calculated molecular weight: 5107.13 Da. Experimental m/z: [M+3H]^3+^: 1703.3770 (****A****); [M+4H]^4+^: 1277.7780 (****B****); [M+5H]^5+^: 1022.7249 (****C****); [M+6H]^6+^: 852.2010 (****D****); [M+7H]^7+^: 730.5889 (****E****).*

**Figure 7S.** Chemical structure of the CF-Tat-N_3_-NH_2_ together with the ESI-MS spectrum. Molecular formula: C_85_H_135_N_37_O_19_, [M+H]^1+^_Calculated_: 1979.1; Experimental mass: [M+H] ^+^: 1977.9, [M+2H]^2+^: 989.8, [M+3H]^3+^: 660.3.

**Figure 8S.** Chemical structure of CF-PNT-Ahx-N_3_-NH_2_ together with the ESI-MS spectrum. Molecular formula: C_135_H_195_N_39_O_28_, [M+H]^1+^_Calculated_: 2813.3; Experimental mass: [M+H]^+^: 2811.6, [M+2H]^2+^: 1405.7, [M+3H]^3+^: 937.6.

**Figure 9S.** Analytical HPLC chromatogram of the Fmoc-RRR-Ahx-C-NH_2_ (**A**) Fmoc-RRR-βAla-C-NH_2_ (**B**) Fmoc-RRR-C-NH_2_ (**C**) crude, after the cleavage, recorded using a Kinetex C18 column, 1mL/min flow rate, with detection at 220nm. Elution was initially performed at 5% of solvent B, followed by a linear gradient from 5% to 95% over 30 minutes. The product peak, if present, is indicated by the arrow.

**Figure 10S.** Chemical structure of the CF-Tat-Ahx-Cys(TNP)-OH together with the ESI-MS spectrum. Molecular formula: C_90_H_137_N_35_O_22_S_2_, [M+H]^1+^_Calculated_: 2125.3; Experimental mass: [M+H]^+^: 2124.9, [M+2H]^2+^: 1062.9, [M+3H]^3+^: 709.0, [M+H]^+#^: 1968.9 represents the peptide experimental mass after the loss of the activation on the Cys residue.

***Figure 11S.*** *Chemical structure of CF-PNT-Cys(TNP)-OH together with the ESI-MS spectrum. Molecular formula: C_140_H_198_N_38_O_30_S_2_, [M+H]^1+^_Calculated_: 2958.2; Experimental mass: [M+H]^+^: 2957.9, [M+2H]^2+^: 1478.8, [M+3H]^3+^: 986.3.*

**Figure 12S.** Chemical structure of CF-Tat(triazole)PNA-NH_2_ together with ESI-MS spectrum. Molecular formula: C_296_H_406_N_142_O_76_, [M+H]^1+^_Calculated_: 7181.5; Experimental mass: [M+3H]^3+^: 2396.1, [M+4H]^4+^: 1795.8.

**Figure 13S.** Chemical structure of CF-PNT(triazole)PNA-NH_2_ together with the ESI-MS spectrum. Molecular formula: C_346_H_467_N_145_O_76_, [M+H]^1+^_Calculated_: 8020.0; Experimental mass: [M+3H]^3+^: 2671.7, [M+4H]^4+^: 2004.0, [M+5H]^5+^: 1603.3.

**Figure 14S.** Chemical structure of CF-Tat-S-S-PNA-NH_2_ together with the ESI-MS spectrum. Molecular formula: C_294_H_406_N_140_O_76_S_2_, [M+H]^1+^_Calculated_: 7181.7; Experimental mass: [M+3H]^3+^: 2396.1, [M+4H]^4+^: 1795.8, [M+5H]^5+^: 1437.0.

**Figure 15S**. Chemical structure of CF-PNT-S-S-PNA-NH_2_ together with the ESI-MS spectrum. Molecular formula: C_344_H_467_N_143_O_84_S_2_, [M+H]^1+^_Calculated_: 8014.4; Experimental mass: [M+3H]^3+^: 2671.7, [M+4H]^4+^: 2004.1, [M+5H]^5+^: 1603.3.

**Figure 16S.** UV-melting curves at 260 nm of miRNA 221-5p at 2.5 µM concentration in 10 mM NaCl, 10 mM phosphate buffer pH=7.4.

**Figure 17S.** UV-melting curves at 260 nm of miRNA 221-5p/scramble duplex at 2.5 µM concentration in 10 mM NaCl, 10 mM phosphate buffer pH=7.4

**Figure 18S.** Cell proliferation assays. CF-Tat-(triazole)-PNA (**A**), CF-Tat-S-S-PNA (**A’**) and. CF-PNT-(triazole)-PNA (**B**), CF-PNT-S-S-PNA (**B’**), were tested on A549 cells at a final concentration of 5 μM, either alone or in combination with calcium ions (Ca^2+^) 5 mM. Cell viability was evaluated after 1h, 24h, and 48h using the CCK-8 assay. The percentage of viable cells is expressed relative to the corresponding control, consisting of cells incubated for the same duration under identical conditions (complete culture medium and serum), but without peptide or additives.
